# Stabilization of Hypoxia-Inducible Factor-1 Alpha Augments the Therapeutic Capacity of Bone Marrow-Derived Mesenchymal Stem Cells in Experimental Pneumonia

**DOI:** 10.3389/fmed.2018.00131

**Published:** 2018-05-04

**Authors:** Naveen Gupta, Victor Nizet

**Affiliations:** ^1^Division of Pulmonary and Critical Care, Department of Medicine, School of Medicine, University of California, San Diego, La Jolla, CA, United States; ^2^Department of Molecular Medicine, The Scripps Research Institute, La Jolla, CA, United States; ^3^Division of Host-Microbe Systems and Therapeutics, Department of Pediatrics, School of Medicine, University of California, San Diego, La Jolla, CA, United States; ^4^Skaggs School of Pharmacy and Pharmaceutical Sciences, School of Medicine, University of California, San Diego, La Jolla, CA, United States

**Keywords:** mesenchymal stem cells, hypoxia-inducible factor-1 alpha, lung injury, pneumonia, sepsis

## Abstract

Bone marrow-derived mesenchymal stem cells (MSCs) have therapeutic effects in experimental models of lung injury. Hypoxia-inducible factor-1 alpha (HIF-1α) is a transcriptional regulator that influences cellular metabolism, energetics, and survival under hypoxic conditions. The current study investigated the effects of stabilizing HIF-1α on the therapeutic capacity of MSCs in an experimental mouse model of bacterial pneumonia. HIF-1α stabilization was achieved by the small molecule prolyl-hydroxlase inhibitor, AKB-4924 (Aerpio Therapeutics, Inc.), which blocks the pathway for HIF-1α degradation in the proteosome. *In vitro*, pre-treatment with AKB-4924 increased HIF-1α levels in MSCs, reduced the kinetics of their cell death when exposed to cytotoxic stimuli, and increased their antibacterial capacity. *In vivo*, AKB-4924 enhanced MSC therapeutic capacity in experimental pneumonia as quantified by a sustainable survival benefit, greater bacterial clearance from the lung, decreased lung injury, and reduced inflammatory indices. These results suggest that HIF-1α stabilization in MSCs, achieved *ex vivo*, may represent a promising approach to augment the therapeutic benefit of these cells in severe pneumonia complicated by acute lung injury.

## Introduction

Severe pneumonia is the most common cause of sepsis and respiratory failure among critically ill patients. The mortality in the most severe cases can approach 50%, and treatment options have become increasingly limited due to the rapid emergence of multi-drug resistant bacterial strains, particularly among enteric Gram-negative bacteria (1–3). New treatment options that can harness the potential of the innate immune system are needed to more effectively manage this complex condition.

Bone marrow-derived mesenchymal stem cells (MSCs) have been studied as a potential source for cell-based therapy for a wide range of experimental organ injury models. In particular, there has been a considerable amount of focus on using MSCs as a therapy for severe lung injury and sepsis as there are no proven pharmacological therapies in this field (4–9). MSCs have a number of biological properties that lend them to producing a favorable outcome in lung injury and sepsis including immunomodulation, secretion of epithelial and endothelial growth factors, and augmentation of host defense to infection (6, 10, 11). However, the clinical benefits of MSCs in trials have been modest, which may be due to a lack of sustained benefit given MSC death and clearance under inflammatory conditions *in vivo*. It has previously been shown that non-viable MSCs exert no therapeutic benefit (5). Thus, methods to enhance MSC survival and augment their therapeutic capacity should improve their efficacy in clinical lung injury and sepsis.

Hypoxia-inducible factor-1 alpha (HIF-1α) is an important transcriptional regulator that controls many cellular processes under hypoxic conditions, and the injured lung represents a low-oxygen tension environment that presents a metabolic stress to cells introduced into that space. Prior efforts suggested that stabilization of cellular HIF-1α levels could increase the therapeutic function of MSCs in cardiac and vascular injury models (12–14). Consequently, we hypothesized that HIF-1α stabilization in MSCs would enhance their therapeutic efficacy in experimental lung injury and pneumonia, potentially by improving cell survival in the face of inflammatory, cytotoxic stimuli. To that end, we pharmacologically stabilized HIF-1α in MSCs using AKB-4924 (Aerpio Therapeutics, Blue Ash, OH, USA) given our previous experience with the selective potency of this compound (15–17).

## Methods

### Isolation, Characterization, and Culturing of MSCs

Mouse MSCs were isolated from 8- to 10-week old male C57BL/6J mice and characterized as published before (8). MSCs were then cultured using MEM-alpha media (Gibco, catalog #12561) with 15% FBS (Gibco, catalog #12662-029) and 1% Pen/Strep/l-Glutamine and used for *in vitro* and *in vivo* experiments from passages 5 to 10.

### HIF-1α Stabilization in MSCs and Western Blotting

Mesenchymal stem cells were incubated in the presence of AKB-4924 in a 12-well plates for 4 and 24 h to determine the optimal time and concentration for HIF-1α stabilization in MSCs. AKB-4924 was used at 10 and 100 µM in MEM-alpha supplemented with 5% FBS. MSCs were then lysed and the protein fraction isolated, quantified, and analyzed for HIF-1α expression by Western blotting (see Supplementary Material for details). Based on the data, AKB-4924 was used at 100 µM for 4 h on MSCs to stabilize HIF-1α in most *in vitro* and *in vivo* studies.

### *In Vitro* Bacterial Killing Studies

To determine if AKB-4924 enhances MSC killing of bacteria, separate assays were done with live MSCs and MSC-derived conditioned media in the presence of *Escherichia coli* (see Supplementary Material). Mouse cathelicidin-related antimicrobial protein (CRAMP ELISA, MyBioSource, catalog #MBS280706) was specifically measured to determine if it accounted for the antimicrobial effects induced by AKB-4924. Gene expression for CRAMP was quantified using qPCR as outlined below.

### *In Vitro* Cell Death and Caspase 3/7 Activity

To measure the effect of AKB-4924 on MSC death when exposed to cytotoxic, inflammatory stimuli, studies were done to measure caspase 3/7 activity in a plate-based assay (Promega, catalog #G7790). TNF-α and cycloheximide were chosen as the stimuli since this combination resulted in the most reproducible quantity of cell death for MSCs, and it has been published as an *in vitro* method to model cell death in an inflammatory environment (18, 19) (see Supplementary Material).

### RNA Isolation and qPCR

*In vitro* studies were done to determine if AKB-4924 regulated expression of selected genes (CRAMP, Oct4, TWIST) in MSCs that could account for the observed *in vitro* and *in vivo* effects. RNA was isolated and qPCR was carried out using standard procedures (see Supplementary Material).

### *In Vivo E. coli* Pneumonia Model and Experimental Design

All mice used for these experiments were male C57BL/6J (Jackson Labs) between the ages of 10 and 15 weeks of age. All experiments were approved by the University of California, San Diego (UCSD) Institutional Animal Care and Use Committee, and mice were housed in a UCSD facility approved by the Association for Assessment and Accreditation of Laboratory Animal Care. The general experimental design that we followed is as previously published (5, 8) (see Supplementary Material).

### Assessment of Lung Injury, Inflammation, and Bacterial Burden

Lung injury was assessed by histological methods and scored using a previously published method (20). Markers of inflammation and permeability were measured in the bronchoalveolar lavage (BAL) fluid (5, 8), and bacterial burden was calculated from whole lung homogenate (see Supplementary Material).

### Statistical Analysis

The majority of the data is presented as mean ± SD for each group analyzed. An unpaired, two-sided Student’s *t*-test was used for comparisons between sets of data. For sets of data with a small sample size (total *n* < 20), a Mann–Whitney *U* test was used. If multiple groups of data were compared simultaneously, an ANOVA was used. Survival data were analyzed using a log-rank test. A *p*-value <0.05 was used for statistical significance for all analyses.

## Results

### AKB-4924 Stabilizes HIF-1α in MSCs and Reduces MSC Death Under Cytotoxic Conditions

**Figure 1:**
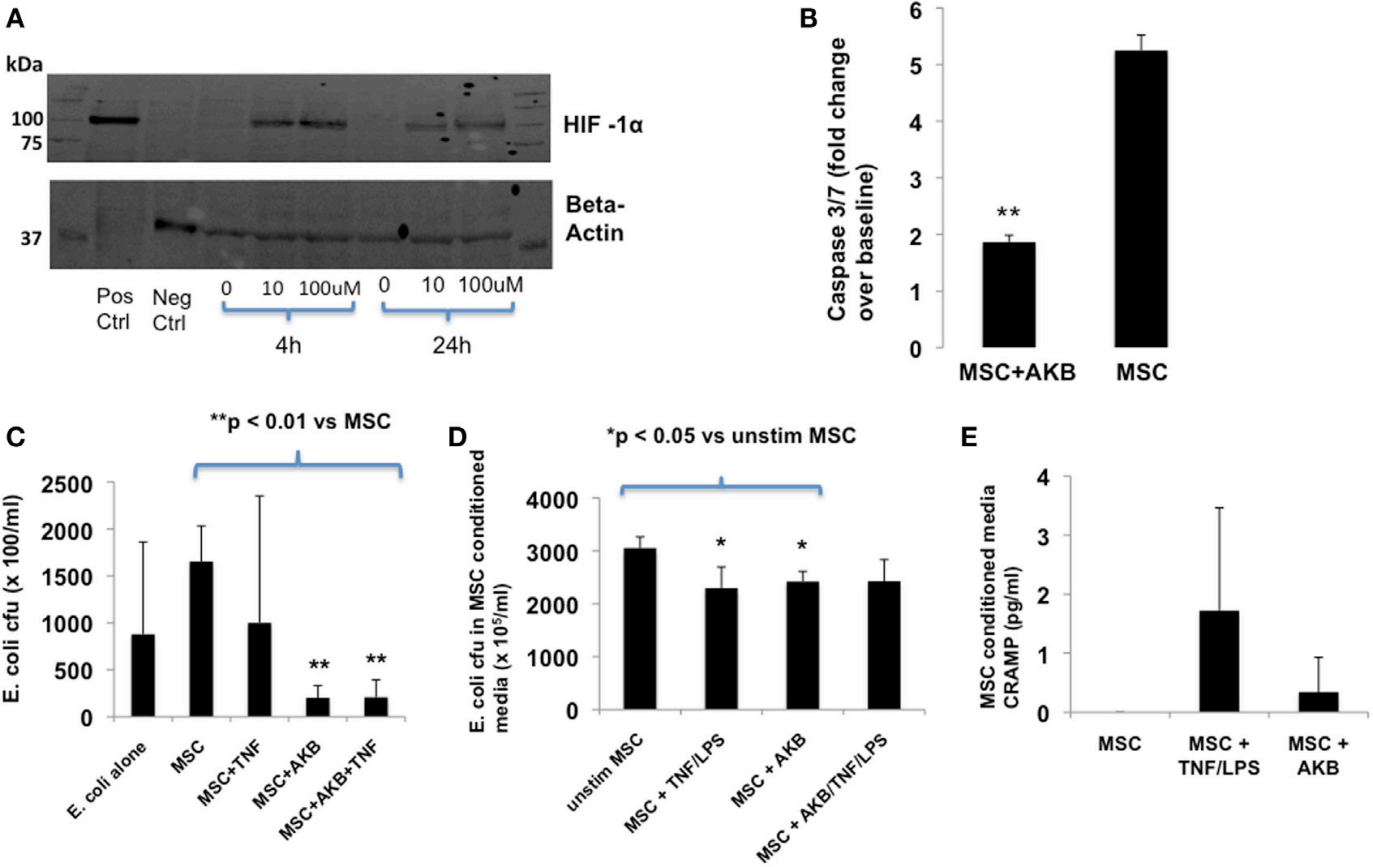
AKB-4924 stabilizes hypoxia-inducible factor 1α (HIF-1α) in mesenchymal stem cells (MSCs) and improves MSC survival and bacterial clearance under *in vitro* conditions. Use of AKB-4924 at both 10 and 100 µM resulted in detectable amounts of HIF-1α protein in MSCs after 4 and 24 h of incubation **(A)**. AKB-4924 significantly reduced caspase 3/7 activity in MSCs exposed to TNF-α and cycloheximide for 5 h [**(B)**, **p < 0.01 for MSC + AKB vs MSC, *n* = 6 per group]. MSCs pre-stimulated with AKB-4924 exhibited enhanced bacterial clearance at 6 h [**(C)**, **p < 0.01 when compared with MSC group, *n* = 4 per group] that may be partially due to a soluble antimicrobial factor [**(D)**, *p < 0.05 compared with unstim group, *n* = 4 per group]. Cathelicidin-related antimicrobial protein (CRAMP) was not significantly increased in the conditioned media of MSCs pre-treated with AKB-4924 when compared with unstimulated MSCs [**(E)**, *n* = 3 per group].

AKB-4924 stabilization of HIF-1α protein levels in MSCs occurred at a concentration of 10 or 100 µM and was readily apparent after 4 h incubation (Figure 1A). MSCs treated with AKB-4924 exhibited significantly reduced cell death, as measured by caspase 3/7 activity, when exposed to TNF-α and cycloheximide (Figure 1B).

### AKB-4924 Enhances the Antibacterial Capacity of MSCs

*In vitro*, AKB-4924 was able to significantly improve MSC-based reduction of viable *E. coli*. The effect of AKB-4924 occurred under both basal and TNF-α stimulated conditions (Figure 1C). Conditioned media from AKB-4924 stimulated and TNF-α + LPS stimulated MSCs demonstrated an approximate 20% reduction in viable *E. coli* compared with conditioned media from unstimulated MSCs (Figure 1D). This suggests that release of an antimicrobial factor into the conditioned media may account for part of the increased bacterial killing by MSCs that is induced by AKB-4924. We hypothesized that this factor may be mouse CRAMP given previous literature demonstrating that the human cathelicidin antimicrobial protein LL-37 is a potential transcriptional target of HIF-1α, and that human MSCs exert antibacterial effects *via* LL-37 secretion (10, 21). However, under the conditions utilized in this study, we did not detect a significant increase in CRAMP protein secretion in HIF-1α stabilized MSCs (Figure 1E).

### AKB-4924 Improves MSC-Derived Therapeutic Capacity *In Vivo*

**Figure 2:**
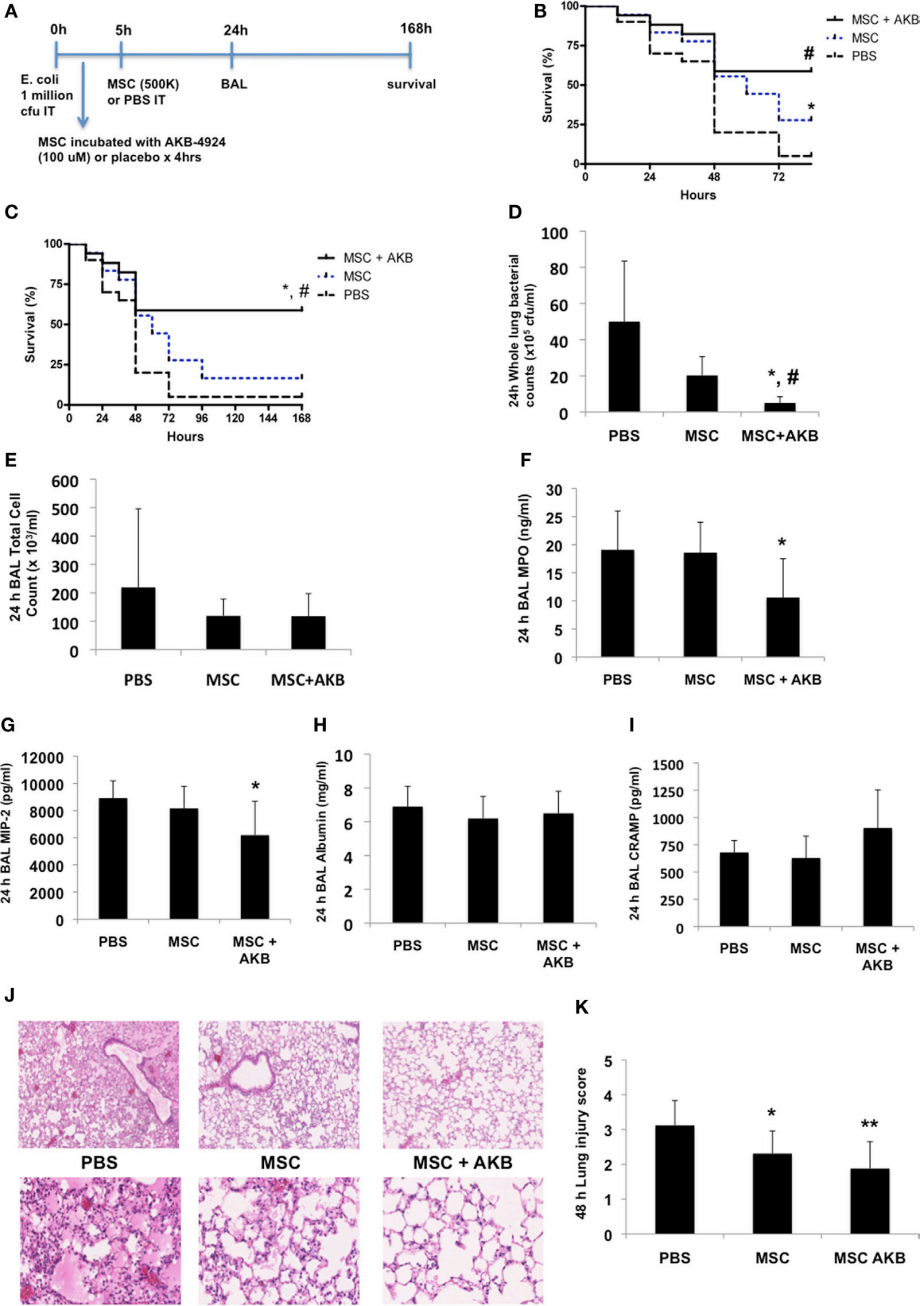
AKB-4924 augments the therapeutic capacity of mesenchymal stem cells (MSCs) in an *Escherichia coli* pneumonia model. Following the experimental design outlined in panel **(A)**, both unstimulated MSCs and MSCs pre-incubated with AKB-4924 (100 µM × 4 h) significantly improved the survival of mice at 72 h [**(B)**, ^#^*p* < 0.05 for MSC + AKB vs PBS, *p < 0.05 for MSC vs PBS, *n* = 17–20 per group], while only AKB-4924 stimulated MSCs increased survival over 7 days [**(C)**, ^*,#^*p* < 0.05 for MSC + AKB vs MSC and PBS treated groups, respectively, *n* = 17–20 per group]. AKB-4924 also significantly improved the ability of MSCs to reduce whole lung bacterial burden [**(D)**, ^*,#^*p* < 0.05 for MSC + AKB vs MSC and PBS treated groups, respectively, *n* = 5–6 per group], alveolar neutrophil influx as measured by bronchoalveolar lavage (BAL) MPO levels [**(F)**, *p < 0.05 for MSC + AKB vs PBS treated group, *n* = 6–12 per group], and inflammation as measured by BAL MIP-2 levels [**(G)**, *p < 0.05 for MSC + AKB vs PBS treated group, *n* = 6 per group]. Total BAL cell counts [**(E)**, *n* = 5 per group], albumin concentration [**(H)**, *n* = 5 per group], and cathelicidin-related antimicrobial protein (CRAMP) levels [**(I)**, *n* = 5 per group] were not significantly changed in the BAL of mice treated with AKB-4924 stimulated MSCs. Lung injury was significantly reduced in both MSC and MSC + AKB treated groups, though the magnitude of improvement was greater in mice treated with AKB-4924 stimulated MSCs [**(J,K)**, *p < 0.05 for MSC vs PBS treated group, **p < 0.01 for MSC + AKB vs PBS treated group, *n* = 8–12 per group; images taken at 2.5 and 20× magnification].

To determine if the *in vitro* benefits with AKB-4924 described above translated into greater MSC-derived therapeutic capacity *in vivo*, the experimental design using an *E. coli* pneumonia model outlined in Figure 2A was utilized. While both unstimulated and AKB-4924 stimulated MSCs exerted significant survival benefits at 72 h (Figure 2B), only MSCs incubated with AKB-4924 conferred sustained protection against mortality over the course of 7 days (Figure 2C). Bacterial clearance from the lung at 24 h post-infection was significantly improved with MSCs incubated with AKB-4924 as well (Figure 2D). In addition, HIF-1α stabilized MSCs led to a significant reduction in inflammatory indices such as BAL myeloperoxidase (MPO) and macrophage inflammatory protein-2 (MIP-2) levels 24 h after infection (Figures 2F,G, respectively), though there was not a significant reduction in the total BAL cell count (Figure 2E) or BAL albumin concentration (Figure 2H). BAL CRAMP was measured to see if it correlated with the reduction in bacterial burden seen in Figure 2D, but the increase in CRAMP observed with HIF-1α stabilized MSCs did not reach statistical significance (Figure 2I). The improvements in bacterial clearance and inflammation were associated with a reduction in lung injury at 48 h post-infection, as assessed by histological methods, that was more pronounced in mice treated with HIF-1α stabilized MSCs (Figures 2J,K).

## Discussion

Mesenchymal stem cells have been extensively studied as a potential therapy for severe lung injury and sepsis and have shown promise in several pre-clinical models (4–11). However, strategies to improve the survival of MSCs in inflammatory environments and thus augment their therapeutic potential are needed. This proof-of-principle study sought to enhance the therapeutic potential of MSCs in experimental lung injury due to pneumonia by stabilizing the transcription factor HIF-1α with the pharmacological agent AKB-4924. Results from this study substantiated our hypothesis by demonstrating that AKB-4924 improved: (a) MSC survival under *in vitro* cytotoxic conditions; (b) MSC antibacterial activity *in vitro*; and (c) MSC-derived therapeutic capacity *in vivo* with reduced mortality, bacterial burden, inflammation, and lung injury. Though, it is interesting to note that while BAL MPO levels were reduced, total BAL cell counts were not in this study. This discordance may due to a greater effect on neutrophil degranulation as opposed to absolute neutrophil recruitment to the alveolar space. Also, the lack of reduction in BAL albumin at 24 h is not concordant with the other parameters measured, which may be because it represents a summation of permeability over the entire time period and is not sensitive enough to detect changes that develop later in the timeframe being studied. Nevertheless, the overall findings suggest that methods to stabilize HIF-1α in MSCs could be implemented in order to boost the therapeutic effect achieved in critically ill patients with lung injury, and are consistent with recent promising results in cardiac and vascular disease models (12–14).

Mesenchymal stem cells have been tested in several hundred clinical trials to date targeting a wide range of clinical diseases, but their clinical efficacy has not been reproducibly robust to date (22, 23). One of the potential explanations that has been suggested is the relatively short half-life of MSCs *in vivo* (24, 25). HIF-1α represents an intuitive target to augment survival of MSCs in lung injury applications since the injured lung is a hypoxic environment requiring metabolic adaptations. Recent studies in experimental models of ischemia-reperfusion and radiation-induced lung injury have shown that hypoxic preconditioning of MSCs enhances their therapeutic efficacy (26, 27). The mechanisms demonstrated include improved MSC survival and antioxidant ability.

In this study, HIF-1α stabilization in MSCs with the use of AKB-4924 resulted in significantly improved MSC survival under cytotoxic conditions and MSC-derived therapeutic capacity *in vivo*. While improving MSC survival is likely an important contributor to the augmented biological effect achieved with HIF-1α stabilized MSCs, there are other possible mechanisms to consider. We provide some preliminary data that HIF-1α stabilization augments the antibacterial property of MSCs, and it is possible that HIF-1α stabilization in MSCs may be boosting other biological effects of MSCs such as growth factor secretion and immunomodulation. We also tested the possibility that HIF-1α stabilization could keep MSCs in an undifferentiated, “stem-like” state that permits them to retain their reparative properties for a longer duration (28). However, screening qPCR analyses to determine if HIF-1α stabilization upregulated-specific genes involved in maintaining an undifferentiated MSC phenotype (Oct4, TWIST) were unable to detect a significant difference compared with unstimulated MSCs (Figure S1 in Supplementary Material). Finally, HIF-1α stabilized MSCs may be modulating the survival and function of other cell types that are known to be present in the injured lung such as alveolar epithelial cells, endothelial cells, neutrophils, and macrophages. These other potential mechanisms remain the focus of ongoing and future investigations.

While we used a small molecule, AKB-4924, to stabilize HIF-1α in MSCs there are other potential methods that could be used to achieve this goal. Previous studies have used hypoxic preconditioning (i.e., growing MSCs under hypoxic conditions) to augment HIF-1α expression. In addition, genetic editing could be applied to MSCs in order to inactivate the prolyl hydroxylase enzymes responsible for HIF-1α degradation under normoxic conditions. However, genetic editing may carry an increased risk of malignant transformation of MSCs due to sustained dysregulation of HIF-1α expression, particularly since HIF-1α has been implicated in tumor development and invasiveness (29–31). In this regard, the use of AKB-4924 affords the advantage of stabilizing HIF-1α for a defined time period that is determined by its own half-life. For acute inflammatory processes, such as lung injury due to bacterial pneumonia, even transient stabilization of HIF-1α can lead to significant beneficial outcomes as we observed.

In summary, stabilization of HIF-1α in MSCs, with the use of AKB-4924, significantly boosts MSC-derived therapeutic capacity in an *E. coli* model of bacterial pneumonia. Mechanistically, this may be due, in part, to improved MSC survival under cytotoxic conditions. This study and other recent publications suggest that strategies to stabilize HIF-1α should be incorporated into MSC-based clinical trials for critically ill patients with lung injury.

## Ethics Statement

All experiments were approved by the University of California, San Diego (UCSD) Institutional Animal Care and Use Committee (IACUC), and mice were housed in a UCSD facility approved by the Association for Assessment and Accreditation of Laboratory Animal Care (AAALAC).

## Author Contributions

NG and VN planned the experiments, wrote the manuscript, and funded the studies. NG carried out the experiments.

## Conflict of Interest Statement

The authors declare that the research was conducted in the absence of any commercial or financial relationships that could be construed as a potential conflict of interest. The reviewer AK declared a past co-authorship with one of the authors NG to the handling Editor.
