# Supplementary material for: Stabilization of Hypoxia-Inducible Factor-1 Alpha Augments the Therapeutic Capacity of Bone Marrow-Derived Mesenchymal Stem Cells in Experimental Pneumonia
Source: Front Med (Lausanne). 2018 May 4;5:131. doi: 10.3389/fmed.2018.00131 (PMC5945808; doi:10.3389/fmed.2018.00131)
Supplement: Supplementary file 1 [file data_sheet_1.DOC]

**Supplementary Material**

**Methods**

*HIF-1α stabilization in MSCs and Western blotting*

MSCs were lysed and the protein fraction isolated using RIPA buffer (ThermoFisher). The protein was quantified using the BCA method (ThermoFisher) and approximately 30 μg of total protein was loaded on to a 4-12% Bis-Tris gel (NuPage, Invitrogen) after being reduced and denatured. Positive and negative controls for HIF-1α (Novus Biologicals, catalog #NB800-PC26) were also loaded, and the gel was run, transferred and blocked using standard conditions. An anti- HIF-1α primary antibody (Novus, catalog #NB100-449) and corresponding secondary antibody (LiCor) were used to detect the HIF-1α protein on the membrane. A LiCor machine was used to develop and visualize the bands.

*In vitro bacterial killing studies*

The *E. coli* K1 bacteria used in these studies was originally isolated from the blood of a patient with biliary sepsis and provided by Xiao Su, MD, PhD (Pasteur Institute-Shanghai).

For the direct killing studies, MSCs were plated at 30,000 cells per well in a 24 well plate, allowed to adhere, and then incubated in the presence or absence of AKB-4924 100 μM for 3 h in RPMI supplemented with 5% FBS. The media was removed from each well, wells washed once with PBS, and then fresh media +/- TNF-α (100 ng/ml, R&D, catalog #410-MT-010) was added for an additional 1 h. The media in each well was removed again, wells washed once with PBS, and subsequently *E. coli* [3000 colony forming units, (cfu)] was added to each well. Plates were incubated at 37^o^C for 6 h, the media in each well mixed to equally distribute the bacteria, and an aliquot from each well was diluted and plated on LB agar plates. Colonies were counted the following day to quantify the bacterial numbers in each condition.

Separate studies were done to determine if AKB-4924 induced the release of a soluble antimicrobial factor by MSCs to account for the killing differences observed. In these studies, MSCs were plated at 100,000 cells per well in a 12 well plate, allowed to adhere, and then incubated +/- AKB-4924 100 μM for 4 h in RPMI (no phenol red, Gibco, catalog #11835-030) + 2% FBS. The media was removed, cells washed once with PBS, and stimulated +/- TNF-α (50 ng/ml) and LPS (1 μM, Sigma, catalog #L6529) in fresh media. After 24 h, the conditioned media from each well was harvested and centrifuged to remove cellular debris. 90 μl of each sample was then mixed with *E. coli* (400 cfu in 10 μl LB Broth) in a 96 well plate and incubated at 37^o^C overnight. The following day, each well was diluted and plated on LB Agar plates, incubated at 37^o^C overnight, and colonies counted the next morning.

*In vitro cell death and Caspase 3/7 activity*

MSCs were plated in full culture media at a density of 10,000 cells per well in a black, clear-bottom 96 well plate (Corning) and allowed to adhere overnight. The following day, MSCs were serum starved using MEM-alpha + 0.5% FBS and incubated with AKB-4924 at 100 μM in 90 μl volume overnight for 18 h. Next, the media from each well was changed to fresh MEM-alpha + 0.5% FBS in order to remove any free AKB-4924, and TNF-α (50 ng/ml) and cycloheximide (20 μg/ml, Sigma, catalog #C7698) were added to make the total volume 100 μl per well. After 4 h of exposure, the caspase 3/7-substrate was added to the wells and fluorescence was measured at different time points using a plate reader.

*RNA isolation and qPCR*

MSCs were plated in full culture media at a density of 100,000 cells per well in a 24 well plate and allowed to adhere overnight. The next day, MSCs were incubated +/- AKB-4924 at 100 μM in RPMI + 2% FBS for 4 h. Cells were then washed once with PBS and stimulated +/- TNF-α (50 ng/ml) and LPS (1 μM) in RPMI + 2% FBS for an additional 4 h. MSCs were lysed and RNA isolated isolated using standard procedures (Qiagen Mini-Kit, catalog #74134), and the purity and quantity were assessed using a Nano-drop system. RNA was reverse transcribed into cDNA (Applied Biosystems), and qPCR for selected genes was carried out using a SYBR Green Master Mix (Applied Biosystems, catalog #4309155) and corresponding qPCR primers (Origene). Reaction mixes were incubated in 96 well qPCR plates and run on a qPCR cycler for analysis (BioRad CFX96).

*In vivo E. coli pneumonia model and experimental design*

Survival experiments, using death as the endpoint, were approved by the UCSD IACUC. In non-survival experiments, mice were euthanized with CO_2_ inhalation and then cervical dislocation. *E. coli* K1 strain was used for all *in vivo* experiments, and the experimental design is as we have previously described [5,8]. To briefly summarize, mice were instilled with 1 million cfu of *E. coli* / 25 μl PBS intratracheally (IT) to induce pneumonia. After 5 hours, mice were given treatment with IT MSCs (500,000 cells / 40 μl PBS), MSCs pre-treated with AKB-4924 (100 μM x 4 h), or PBS control (40 μl). Mice were then followed for 24 hours – 7 days to collect samples for analysis and measure survival.

*Assessment of lung injury, inflammation and bacterial burden*

To assess lung injury, mouse lungs from the different treatment groups were inflated and fixed with 10% zinc formalin (ensuring a distention pressure less than 25 cm H_2_O, in order to avoid injury), removed from the thoracic cavity and then immersed in the same fixative solution. Lungs were subsequently embedded in paraffin, sectioned, and stained with hematoxylin and eosin. Images were obtained with a slide scanner (UCSD Histology Core, Hamamatsu Nanozoomer 2.0HT), and lung injury was scored and quantified using a previously published method [20].

Lung inflammation was determined by performing a bronchoalveolar lavage (BAL) in order to measure neutrophil influx and cytokine levels. BAL was done as previously published [5,8], neutrophil influx was quantified by measuring myeloperoxidase (MPO) levels (Invitrogen, catalog #EMMPO), and the cytokine macrophage inflammatory protein-2 (MIP-2) was measured using an ELISA kit (R&D, catalog #MM200). Total cell counts in the BAL were measured using an automated cell counter (Millipore Sigma, Sceptor 2.0). Permeability was assessed by measuring BAL albumin concentration using a BCA protein assay (ThermoFisher, Pierce).

Lung bacterial burden was determined by harvesting the lungs, homogenizing them in PBS and then diluting the homogenate in PBS before plating on LB agar plates. Plates were kept at 37^o^C overnight and cfu were counted the following day to calculate the bacterial burden per lung.

**Results**

**Supplementary Figure 1.**

**
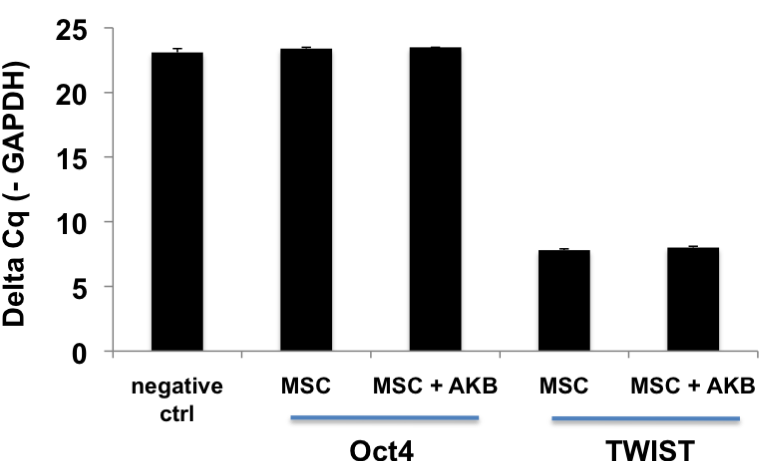
**

**Figure Legend. AKB-4924 does not alter gene expression for Oct4 or TWIST in MSCs.** Gene expression is presented relative to the housekeeping gene, GAPDH, to obtain the delta Cq (a negative control without cDNA was included). AKB-4924 was used at 100 μM for 4 h (n = 4 per group).
